# Supplementary material for: Genome assembly and chemogenomic profiling of National Flower of Singapore Papilionanthe Miss Joaquim ‘Agnes’ reveals metabolic pathways regulating floral traits
Source: Commun Biol. 2022 Sep 15;5:967. doi: 10.1038/s42003-022-03940-6 (PMC9477820; doi:10.1038/s42003-022-03940-6)
Supplement: Supplementary file 2 — Supplementary Information [file 42003_2022_3940_MOESM2_ESM.pdf]

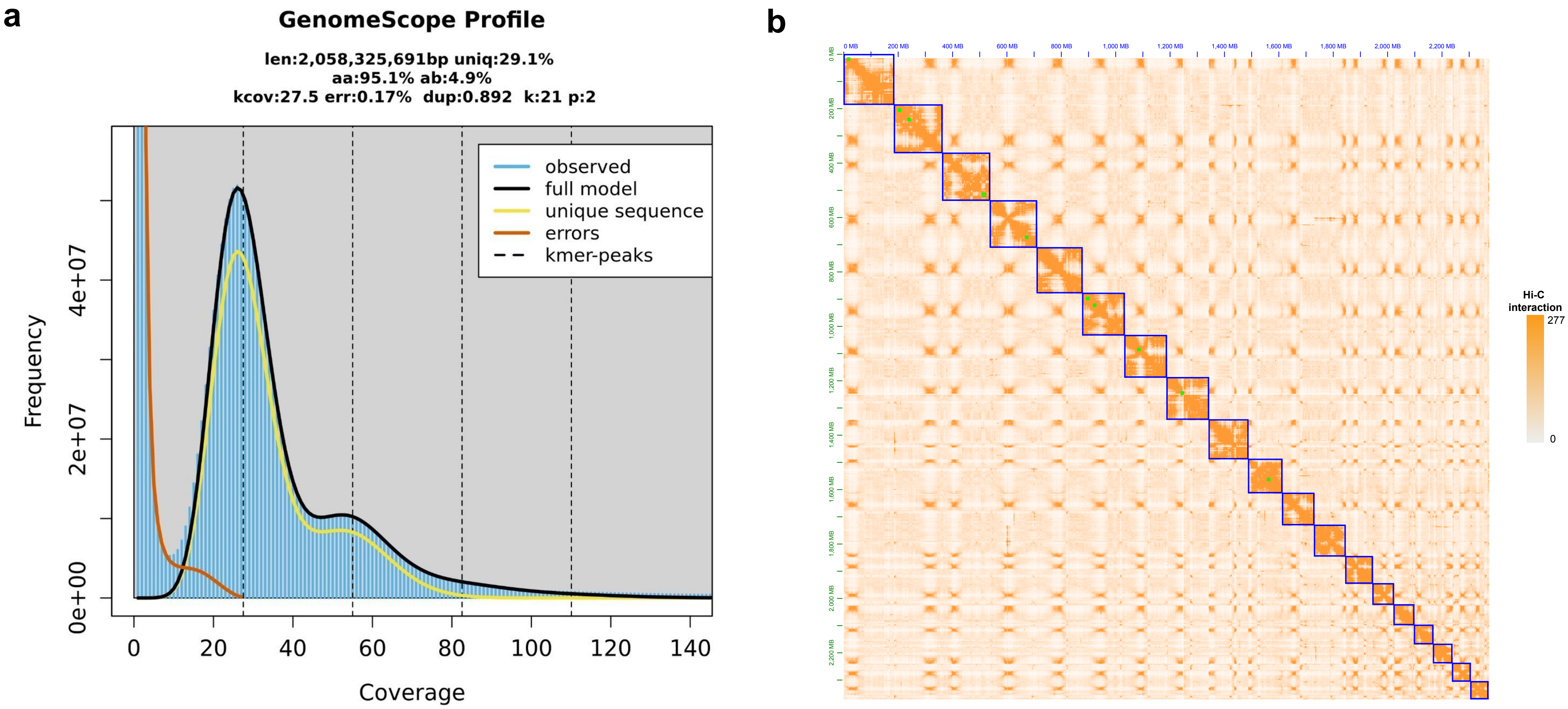

Supplementary Figure 1: Genome of *Paplionanthe* Miss Joaquim 'Agnes'

a Genome survey of PMJ using GenomeScope Profile

b Dovetail Omni-C chromatin interation post scaffolding showing linkages across 19 chromosomal scaffolds visualized using Juicebox.

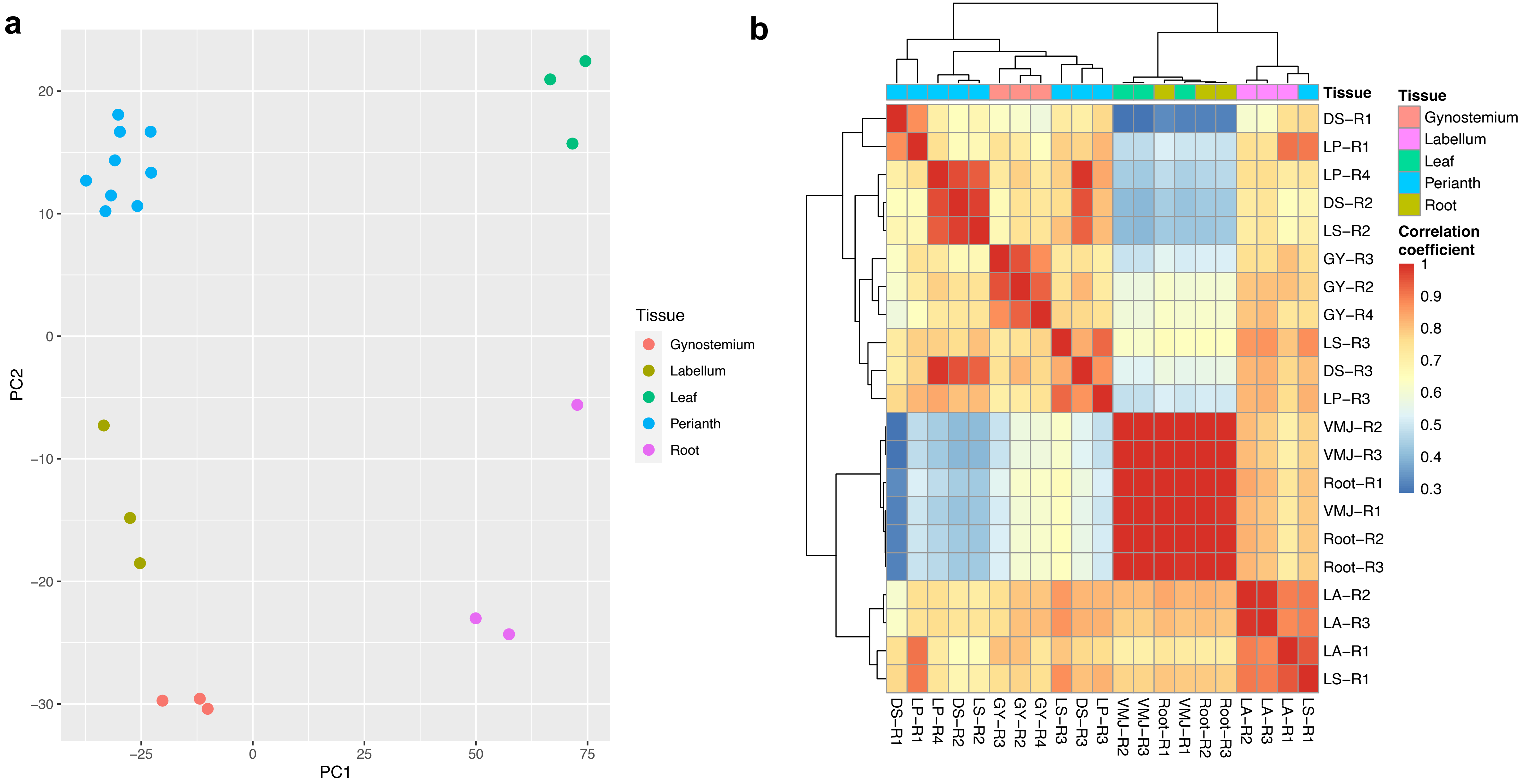

Supplementary Figure 2: RNA-Seq libraries prepared from the tissues of PMJ

a PCA plot of RNA-Seq libraries showing tissue specific clustering of RNA-Seq libraries for the first two principal components.

b Correlation plot with hierarchical clustering of RNA-Seq libraries

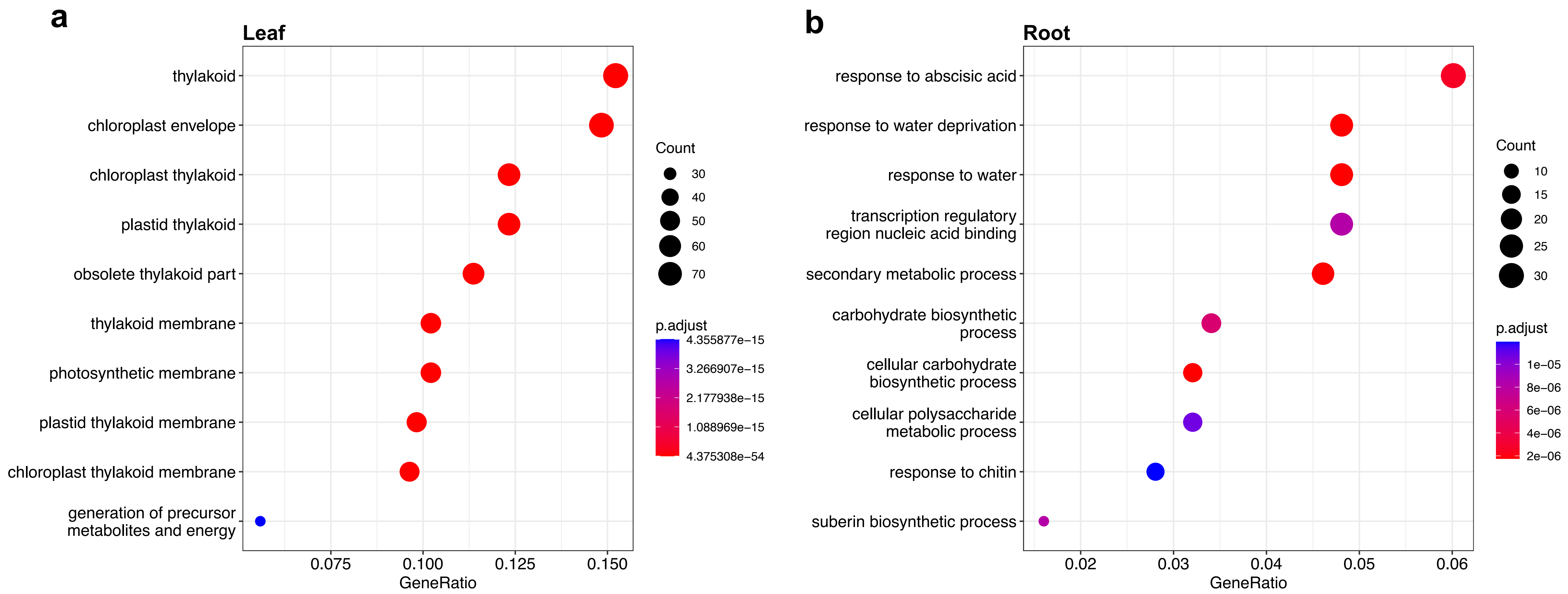

Supplementary Figure 3: GO pathway enrinchment across tissues of PMJ using tissue specific markers from (a) Terete Leaf (b) Root

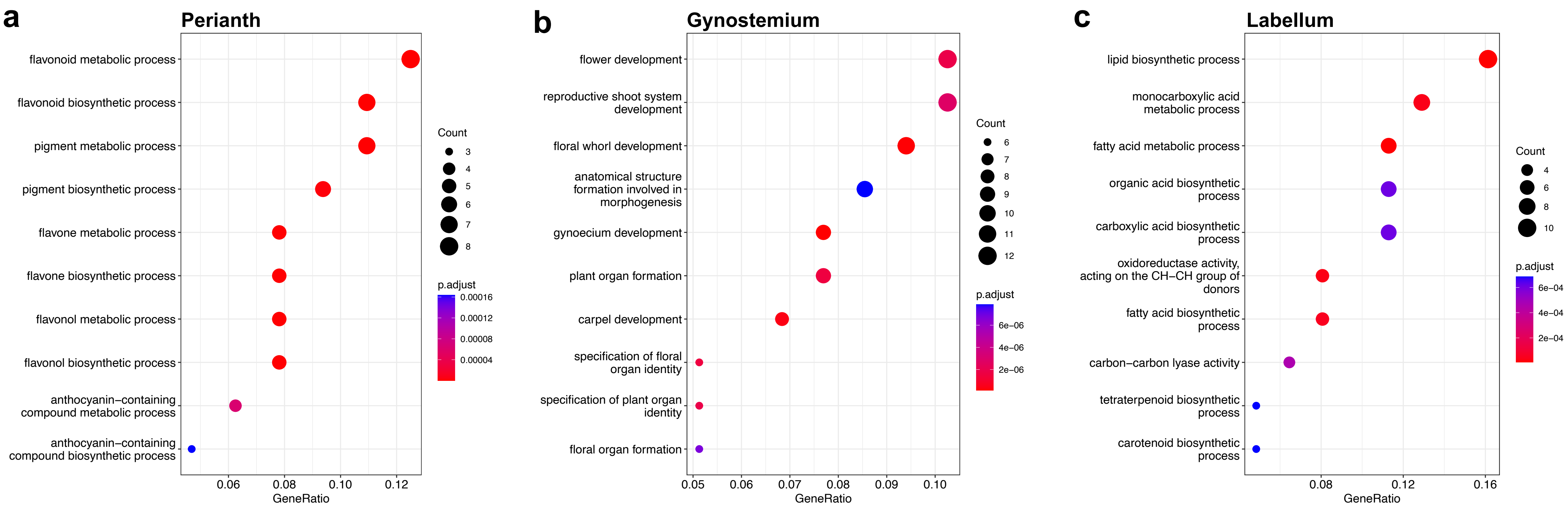

Supplementary Figure 4: GO pathway enrinchment across floral tissue of PMJ using tissue specific markers from (a) Perianth (b) Gynostemium (c) Labellum

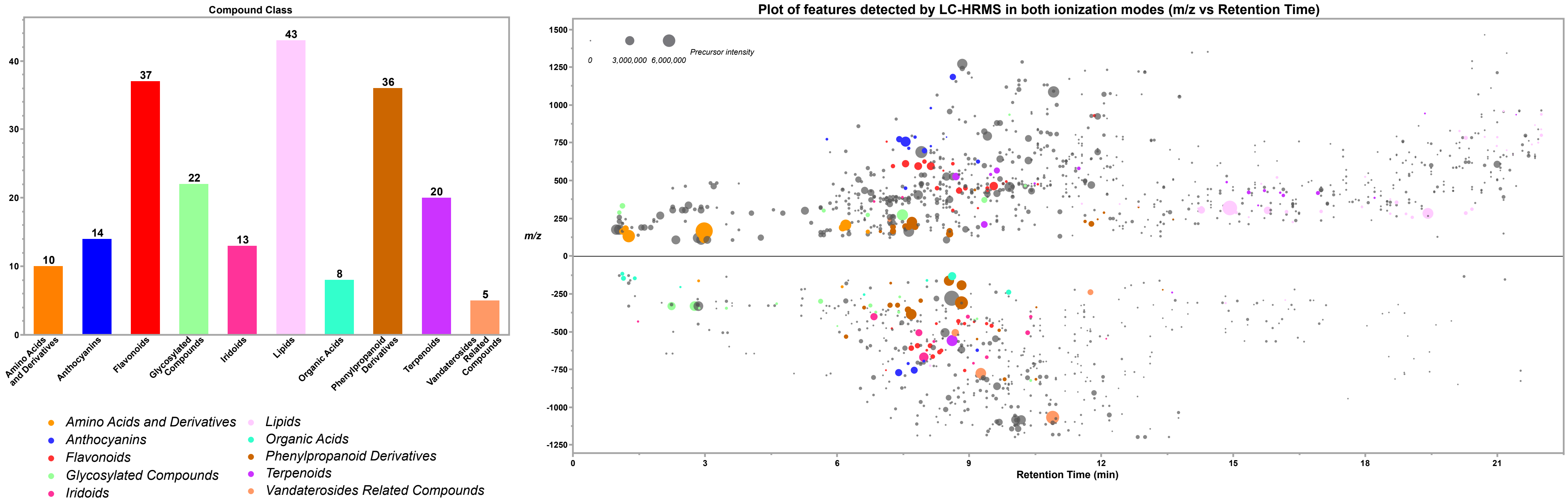

Supplementary Fig 5: Features detected by LC-HRMS in flower and leaves of *Ple*. Miss Joaquim 'Agnes'

a. Bar chart indicating the number of features detected by LC-HRMS across both ionization modes

b. Features detected by LC-HRMS in both positive (top) and negative (bottom) modes in the flower and leaves

a

Color	Compound
Green (chlorophyll)	Pheophytin-a (degradation product of chlorophyll a)
Green (chlorophyllide)	Phaeophorbide b (degradation product of chlorophyllide
Yellow	Apigenin 7-O-(6''-O-acetylglucoside)
Yellow	Apiin (Apigenin 7-O-[beta-D-apiosyl-(1->2)-beta-D-
Yellow	Cosmosin (Apigenin 7-O-beta-D-glucoside)
Yellow	Astragalin (Kaempferol 3-O-glucoside)
Yellow	Astragalin 7-rhamnoside
Yellow	Kaempferin (Kaempferol 3-O-alpha-L-rhamnoside)
Yellow	Kaempferol 3-rhamnoside-7-glucoside
Yellow	Kaempferol 3-sophoroside 7-rhamnoside
Yellow	quercetin
Yellow	Quercetin 3-O-(6''-malonyl-glucoside) 7-O-glucoside
Yellow	Quercetin 3-O-rhamnoside 7-O-glucoside
Yellow	Kaempferol 3-O-beta-D-glucosyl-(1->2)-beta-D-glucoside
Yellow	Trifolin (Kaempferol-3-O-galactoside)
Yellow-orange	Quercitrin
Yellow-orange	isorhamnetin (Quercetin 3'-methyl ether)
Yellow-orange	Isorhamnetin 3-rhamnoside
Yellow-orange	Isorhamnetin 3-rhamnoside-7-glucoside
Yellow-orange	Isorhamnetin 7-glucoside
Yellow-orange	Quercetin 3,3'-dimethyl ether 7-glucoside
Reddish purple	Cyanidin 3-(6''-malonylglucoside)-5-glucoside
Reddish purple	Cyanidin 3-(6-malyl glucoside) 5-glucoside
Reddish purple	Cyanidin 3-(6''-sinapylsophoroside)-5-glucoside
Reddish purple	Cyanidin 3-(disinapoylsophoroside) 5-glucoside
Reddish purple	Cyanidin 3-glucoside 5-caffeoylglucoside
Reddish purple	Cyanidin 3-O-beta-D-glucoside 5-O-(6-coumaroyl-beta-D-
Reddish purple	Cyanidin 3-O-glucoside
Reddish purple	Cyanidin 3-sophoroside-5-glucoside
Magenta	Peonidin 3,5-diglucoside
Magenta	Peonidin 3-sophoroside 5-glucoside

b

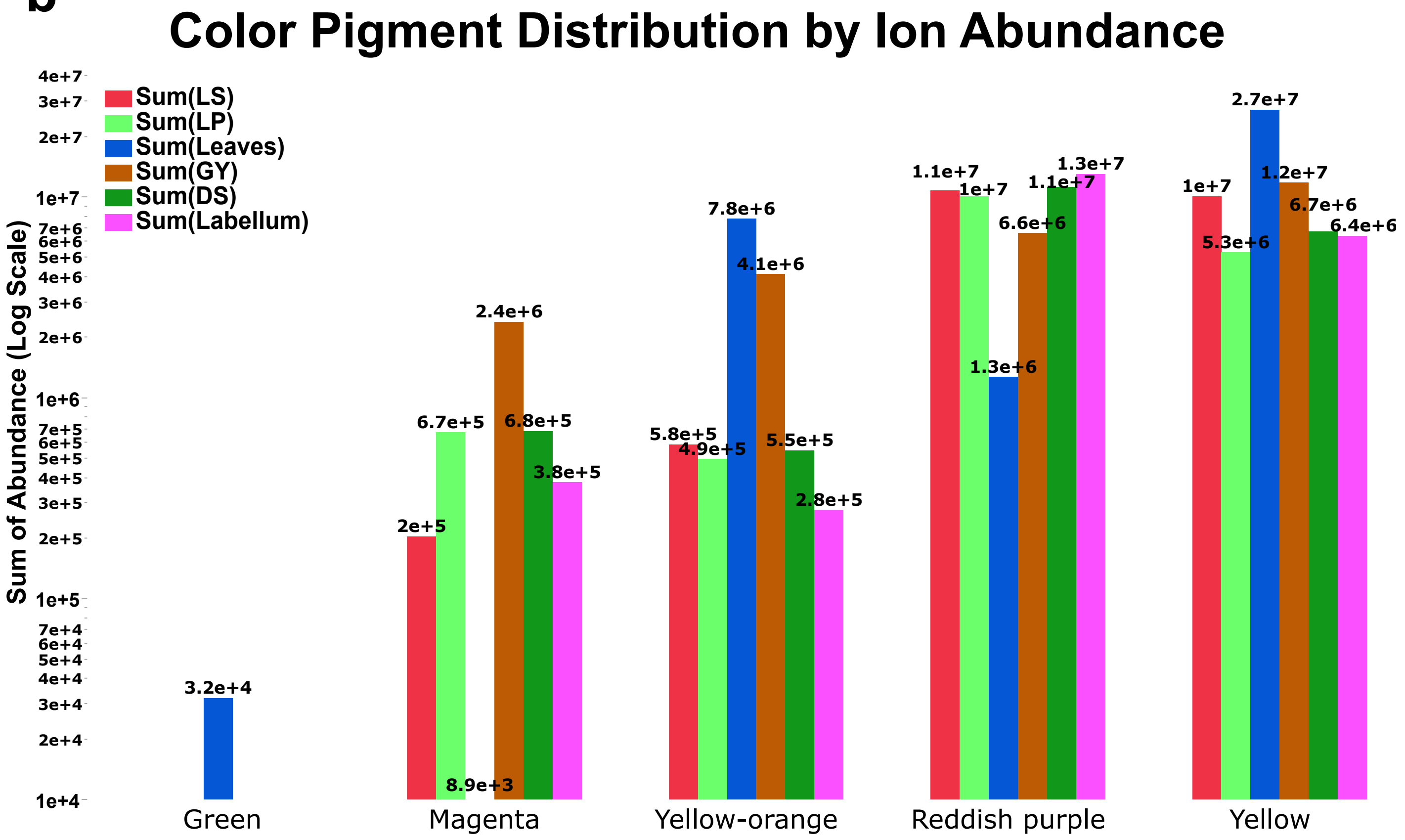

Supplementary Figure 7 : Compounds of anthocyanin derivatives and their abundance in PMJ

a Table showing the associated colors of metabolites detected in the flowers and leaves of PMJ.

b Color pigment distribution aggregrated through the associated abundance of metabolites detected and their associated color across the various leaf and floral tissues of PMJ.

(LS = Lateral Sepal , LP = Lateral Petal, GY= Gynostemium , DS = Dorsal Sepal)

Flower

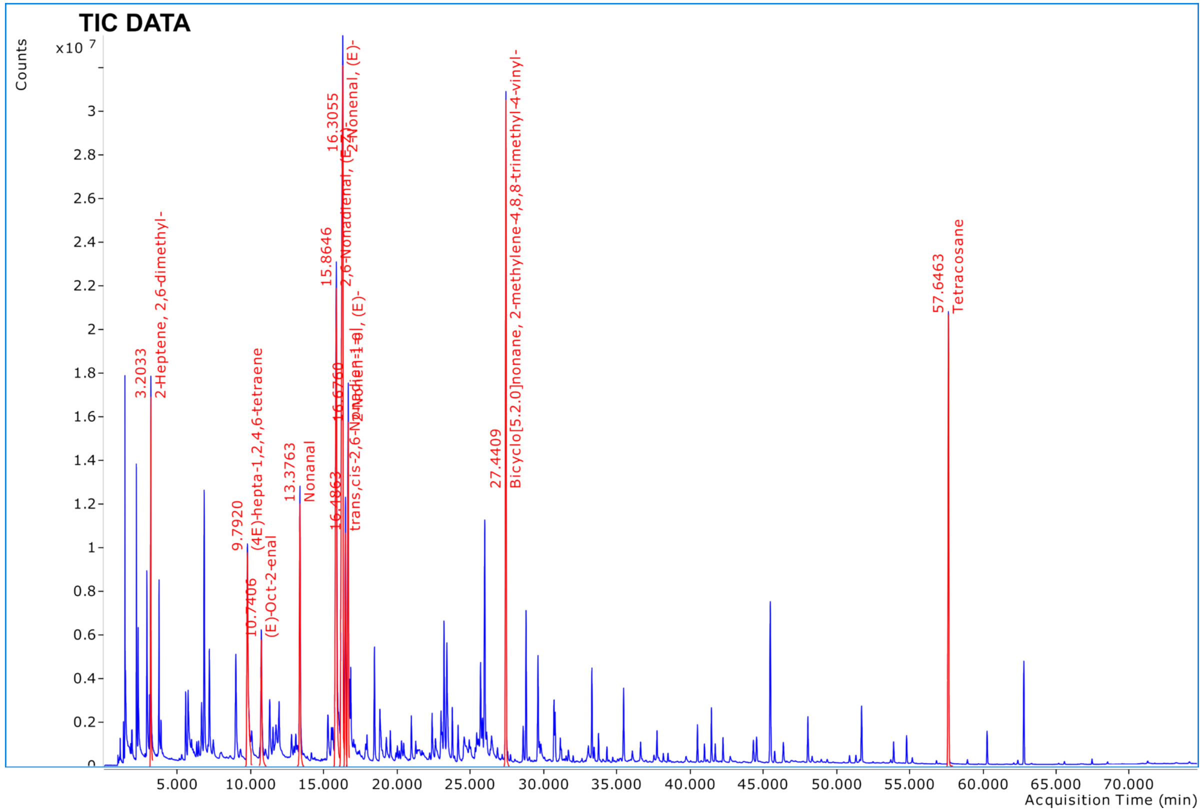

Terete leaf

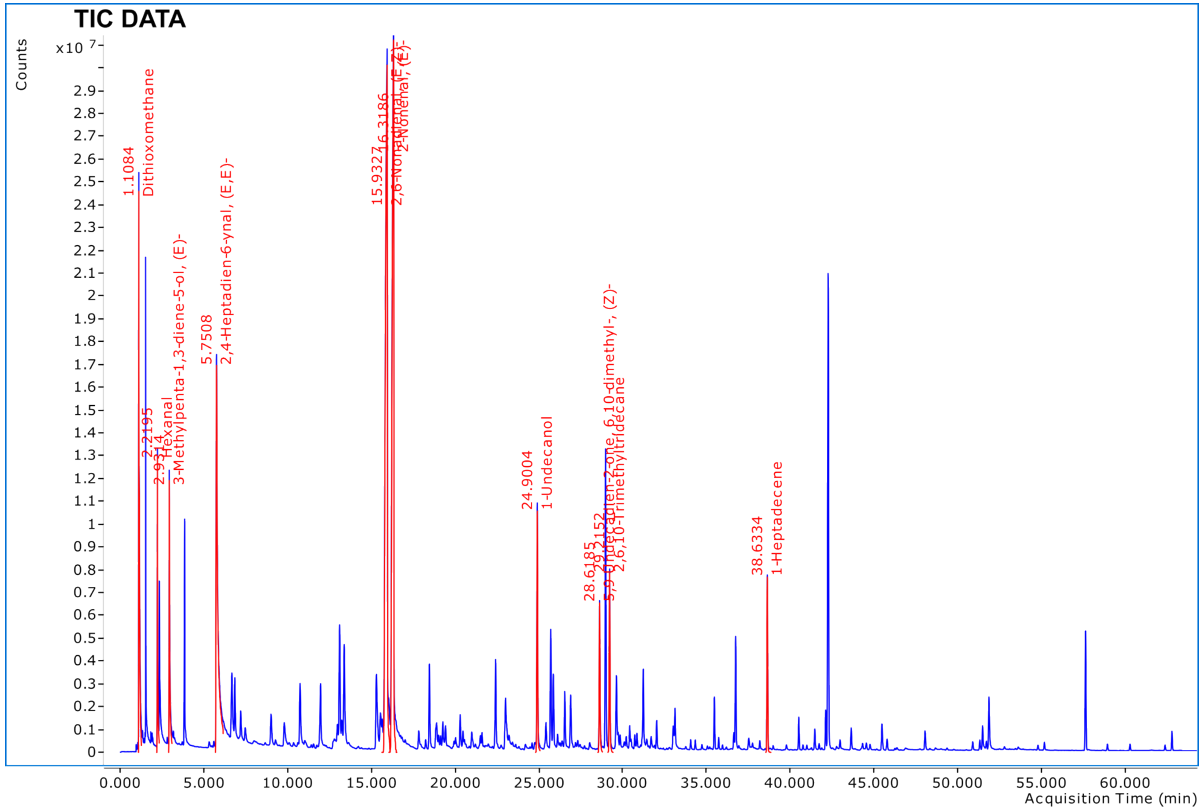

Supplementary Figure 6: GC-HRMS chromatogram for PMJ volatiles in the flower and terete leaf of *Ple* Miss Joaquim ‘Agnes’
