# Supplementary material for: Genome assembly and chemogenomic profiling of National Flower of Singapore Papilionanthe Miss Joaquim ‘Agnes’ reveals metabolic pathways regulating floral traits
Source: Commun Biol. 2022 Sep 15;5:967. doi: 10.1038/s42003-022-03940-6 (PMC9477820; doi:10.1038/s42003-022-03940-6)
Supplement: Supplementary file 3 — Description of Additional Supplementary Files [file 42003_2022_3940_MOESM3_ESM.pdf]

Description of Additional Supplementary Files

File name: Supplementary Data 1

Description: Assembly Scaffold size post proximity ligation scaffolding

File name: Supplementary Data 2

Description: Genome Assembly Stats

File name: Supplementary Data 3

Description: Repeat elements masked by Repeat Masker

File name: Supplementary Data 4

Description: Duplicated genesets in PMJ

File name: Supplementary Data 5

Description: MADS-box TF Genes in Ple. Miss Joaquim 'Agnes'

File name: Supplementary Data 6

Description: Enzymes involved in Anthocyanin Pathway

File name: Supplementary Data 7

Description: Enzymes involved in Stillbenoid/Phenylpropanoid Pathway

File name: Supplementary Data 8

Description: Curated volatiles identified using GC-HRMS in positive/negative mode

File name: Supplementary Data 9

Description: Curated Volatile identified using GC-HRMS and their smell profile

File name: Supplementary Data 10

Description: Beta-ionone synthesis via the carotenoid pathway

File name: Supplementary Data 11

Description: ONT Library Statistics

File name: Supplementary Data 12

Description: Shotgun WGS Library Statistics

File name: Supplementary Data 13

Description: Omni-C Library Statistics

File name: Supplementary Data 14

Description: RNA-Seq Library Statistics

File name: Supplementary Data 15

Description: Source data behind Figure 5b

File name: Supplementary Data 16

Description: Source data behind Figure 6a

File name: Supplementary Data 17

Description: Wilcoxon pairwise test across tissue group for Figure 6a (* p <= 0.05, ** p <0.01)

File name: Supplementary Data 18

Description: Source data behind Fig. 6b

File name: Supplementary Data 19

Description: Source data behind Figure 8d

File name: Supplementary Data 20

Description: Wilcoxon pairwise test across tissue group for Figure 8d (* p <= 0.05, ** p <0.01)
